# Books and Magazines

**Published:** 1912-03

**Authors:** 


### Books and Magazines.

The Taylor Pocket Case Record. By J. J. Taylor, M. D. 252 pages, tough bond paper; red limp leather, $1.00. Published by the Medical Council Co., Forty-second and Chestnut Streets, Philadelphia, Pa.

The object of this book is to encourage more accurate observation and study of cases by supplying a convenient form for a condensed record of each important case, in pocket size, so that the practitioner can have it always with him, and so arranged that the necessary data can be written down in the briefest possible time—preferably while the examination is actually being made.

Thoroughness of examination is encouraged by means of a syllabus, detailing all the points that should be considered in each case.

The blank for the first thorough examination, diagnosis and treatment is followed by spaces for sixteen subsequent visits.

The book provides for 120 cases. It is on a totally different plan from anything heretofore undertaken.

Case Histories in Neurology.—A selection of histories setting forth the diagnosis, treatment and post mortem findings in nervous diseases. By E. W. Taylor, M. D., A. M., Instructor in Neurology, Harvard Medical School, Assistant Physician Department Neurology, Massachusetts General Hospital, etc. Boston, 1911. W. M. Leonard, Publisher. Cloth, 300 pages. Price $3. Illustrated.

The object of this book is to set forth in practical form, on the basis of the Case System, certain fundamental facts regarding the symptomatology, diagnosis, treatment and pathological findings in the more frequent disorders of the nervous system. To accomplish this end, actual cases illustrating definite disease processes or predominating symptoms are narrated in some detail, followed by such explanatory remarks as the individual case demands. Attention has also been given to the important matter of differential diagnosis. The arrangement of the cases has followed the time-honored and useful if somewhat inaccurate anatomical method of division into (1) peripheral, (2) spinal cord, and (3) brain diseases, followed by (4) those for which a definite anatomical basis has not yet been found, and (5) by affections characterized by disorders of function, the neuroses. Very brief explanatory sections on principles of diagnosis and treatment precede and follow the main divisions of the subject matter, which in no way interferes with the primary object of presenting to the reader case histories on the principle developed for medicine by Prof. W. B. Cannon and later put into practical operation by many teachers in Boston and elsewhere.

The book is a broad and carefully prepared clinical course in neurology, dealing with actual cases and supplementing in a way that makes the book not only one of great value but also very interesting, the teachings of the general systematic text-book.

---

Blair's Pocket Therapeutics (a book on a new plan)—A Practitioner's Handbook of Medical Treatment. By Thomas S. Blair, M. D., Neurologist to Harrisburg, Pa., Hospital; author of "A System of Public Hygiene," "Blair's Practitioner's Handbook of Materia Medica," member of the Harrisburg Academy of Medicine, American Medical Association, etc.; 373 pages, special Bible paper; bound in limp leather; price, $2.00. Published by The Medical Council Co., Forty-second and Chestnut Streets, Philadelphia, Pa.

The physician very frequently needs, for instant reference, a book which gives the best methods of treatment in any given case. Many books have been offered for this purpose, but they consisted only of collections of miscellaneous prescriptions and formulas, totally unrelated to each other, with no rules or reasons to guide in their use, and almost useless to the physician with any independence of thought or scientific bent of mind.

This book gives a condensed intelligent discussion of the best methods of treatment, based on scientific principles, with a well-tried, reliable formula occasionally to illustrate the application of the principles. The author gives many modes of treatment far in advance of the present text-books. An ingenious method of indicating relative dosage is to print the name of the drug in capital letters for large doses, in ordinary type for medium doses, and in *italics* for small doses. An exhaustive "Table of Large, Medium and Small Doses" is given in the book.

The diseases treated are divided into related groups, each group occupying a chapter, according to the following classification; a copious alphabetical index provides for instant reference to any particular disease.

The Appendix gives very many necessary tables for quick reference, followed by an exhaustive Table of Doses, closing with a General Index.

In order to get all this within the compass of a book for the pocket, a very thin, tough Bible paper has been used, so that it is really a much larger book than it looks.

This book will be a useful pocket companion to the physician in his daily work.

---

The Practical Medicine Series, 1911.—Comprising Ten Volumes on the Year's Progress in Medicine and Surgery. Under the general editorial charge of Gustavus P. Head, M. D., Professor of Laryngology and Rhinology, Chicago Post-Graduate Medical School. Charles L. Mix, A. M., M. D., Professor of Physical Diagnosis in the Northwestern University Medical School. Volume X. Nervous and Mental Diseases. Edited by Hugh T. Patrick, M. D., Professor of Neurology in the Chicago Policlinic; Clinical Professor of Nervous Diseases in the Northwestern University Medical School; ex-President Chicago Neurological Society. Peter Bassoe, M. D., Assistant Professor of Nervous and Mental Diseases, Rush Medical College. Volume IX, Section 1. Skin and Venereal Diseases. By Wm. L. Baum, M. D., Professor of Skin and Venereal Diseases, Chicago Post-Graduate Medical School. Section 2. Miscellaneous Topics. By Harold D. Moyer, M. D. Price for the set of ten, $10; for either of these two volumes, $1.25. Year-Book Publishers, 180 North Dearborn Street, Chicago.

The miscellaneous topics are: "History of Medicine," "Insurance," "Medico-Legal Questions," "Sociology" and "Miscellaneous." This is alone worth the price of the volume. This work is too well known to now require commendation.

---

Blakiston's Quiz Compends.—A Compend of Genito-Urinary Diseases and Syphilis; Including Their Surgery and Treatment. By Chas. S. Hirsch, M. D., formerly Assistant in the Genito-Urinary Surgical Department, Jefferson Medical College Hospital; Consulting Physician Social Service Hospital and Juvenile Protective Association, Philadelphia. Second edition, with 74 illustrations. Philadelphia: P. Blakiston's Son & Co. 1912. Net price, $1.25.

In this edition the author has carefully revised the entire work, and added considerable new matter, the object being to present, throughout, the subject as complete as possible; at the same time making it thoroughly adaptable for the use of students, and for reference by the practitioner. A number of new cuts have been added; notably that showing the pathological lesions of the verumontanum, and, another, an original dissection of the prostate and seminal vesicles by Barnett. These two subjects have been given considerable attention, owing to the recent contributions made in the diagnosis and treatment of diseases of these structures. The use of bacterins, epididymotomy, vasotomy, anastomosis of the vas deferens for the cure of sterility, fulguration treatment of papillomata of the bladder, use of the Buerger urethrocystoscope, and the phenol-sulpho-naphthalin test, have been included.

---

PRACTICAL ELECTRO-THERAPEUTICS AND X-RAY THERAPY. By J. M. Martin, M. D., Professor of Electro-Therapeutics and X-Ray Methods, Medical Department, Baylor University, Dallas, Texas. Octavo, 480 pages, with 230 engravings. Cloth, $4.00. C. V. Mosby, Medical Book and Publishing Co., St. Louis. 1912.

Here is a splendid book by a Texas author, of which Texas physicians should be proud. It is most creditable, and is gotten out by the Mosbys in fine style. Dr. Martin handles his subject in masterly manner, and presents it in a pleasing and convincing way. This great aid to diagnosis and treatment—electricity—has been too long neglected; it has heretofore been monopolized by the quacks, and so identified with the advertising "doctor" that many have not been disposed to even investigate it; they have given it a black eye. Dr. Martin's book is the first and only really full, intelligent and complete treatise on the subject of which I have any knowledge. I commend it to the earnest attention of my readers as more than "worth while"; it is a necessity. One thing I like about it is the author does not assume, as most writers do, that readers are fully informed in the elements of the subject and that they understand all the terminology, but he goes into a detailed description of such terms as "ohm," "watt," "ampere," etc., and I'll venture 50 per cent of medical men do not know what they mean. The author thus makes clear his subject before he pitches into it. He even attempts to explain what electricity is, and there he is up against it, for no one knows what it is, and one good guess is about as good as another. He selects the guess that it is "ions,"—"electrical units"—the "basis of all matter," etc.

The tall doctor is to be congratulated on the excellence of his book.

I copy the following from the publisher's announcement:

"DRUGLESS THERAPY IS GAINING GROUND EVERY DAY.—Never before has the scientific practitioner of medicine been as hungry for information that will aid him in assisting nature to effect a cure without the deadly drastic depletion to the system which often follows the use of drugs.

"No other therapeutic aid is so beneficial, where properly applied, as electricity, and yet none has been so little understood, improperly used, and abused. We have just now entered the threshold of the wonderful possibilities in the use of this efficient agent in the diagnosis and treatment of disease.

"This work has been written by a practical man, one who has gone through every step in the use of electricity in diagnosis and treatment. He gives the modern conception of what electricity is and what it does—he has tested every electrical device known to modern science. The one aim of the book is to give to the general practitioner the knowledge which will enable him to successfully use electricity in a practical way."

